# Excessive daytime sleepiness in secondary chronic headache from the general population

**DOI:** 10.1186/s10194-017-0794-2

**Published:** 2017-08-16

**Authors:** Espen Saxhaug Kristoffersen, Knut Stavem, Christofer Lundqvist, Michael Bjørn Russell

**Affiliations:** 1 Head and Neck Research Group, Research Centre, Akershus University Hospital, PO Box 95, 1478 Lørenskog, Norway; 2 Department of General Practice, Institute of Health and Society, University of Oslo, Oslo, Norway; 3 Institute of Clinical Medicine, Campus Akershus University Hospital, University of Oslo, Oslo, Nordbyhagen Norway; 4 Department of Pulmonary Medicine, Medical Division, Akershus University Hospital, Lørenskog, Norway; 5 HØKH, Research Centre, Akershus University Hospital, Lørenskog, Norway; 6 Department of Neurology, Akershus University Hospital, Lørenskog, Norway

**Keywords:** Epworth sleepiness scale, Sleep, Posttraumatic headache, Rhinosinusitis, Cervicogenic, Migraine, Population-based

## Abstract

**Background:**

Excessive daytime sleepiness (EDS, defined as Epworth sleepiness scale score > 10) is a common symptom, with a prevalence of 10–20% in the general population. It is associated with headache and other chronic pain disorders. However, little is known about the prevalence of EDS among people with secondary chronic headaches.

**Findings:**

A total of 30,000 persons aged 30–44 from the general population was screened for headache by a questionnaire. The 633 eligible participants with self-reported chronic headache were interviewed and examined by a headache specialist who applied the International Classification of Headache Disorders with supplementary definitions for chronic rhinosinusitis and cervicogenic headache. A total of 93 participants had secondary chronic headache and completed the ESS.

A total of 47 participants had chronic post-traumatic headache (CPTH) and/or cervicogenic headache (CEH), 39 participants had headache attributed to chronic rhinosinusitis (HACRS), while 7 had other secondary headaches. 23.3% of those with CPTH, CEH or HACRS reported EDS. In multivariable logistic regression analysis the odds ratios of EDS were not significantly different in people with CPTH/CEH or HACRS.

**Conclusion:**

Almost one out of four subjects with secondary chronic headache reported EDS with no differences between the various secondary chronic headaches.

## Introduction

Headache and sleep complaints are prevalent in the general population and often coexist in the same subject [1]. Excessive daytime sleepiness (EDS) is associated with neurological disorders and pain [2, 3]. Only a few studies have investigated EDS in headache [4–10], and the results are not uniform, possibly due to differences in methods and patient populations [4–10]. We have previously reported on the prevalence of EDS in primary chronic headaches in the general population [11].

To our knowledge EDS has not been evaluated in people with secondary chronic headache. Thus, in the present study we investigated the prevalence of, and factors associated with EDS in participants from the general population with different secondary chronic headaches.

## Findings

### Methods

#### Study design, population and variables

This was a cross-sectional epidemiological survey of 30,000 representative persons aged 30–44 drawn from the general population of eastern Akershus County, Norway. A postal questionnaire screened for possible chronic headache (≥15 days/last month and/or ≥180 days/last year). Screening-positive subjects were invited to a clinical interview at Akershus University Hospital.

In total 71% (20,598/28,871) of the study population responded to the screening questionnaire. Of 935 patients with self-reported chronic headache, 633 participated in clinical interviews (490 as an ambulatory visit, 143 by telephone). The method has been described in detail elsewhere [12, 13].

After the interview, the participants filled in a self-administered questionnaire including the Epworth Sleepiness Scale (ESS) [14]. The participants also provided information on socio-demographics, height, weight, smoking status, medication-overuse, headache frequency and headache disability by Migraine Disability Assessment (MIDAS) [15].

#### Headache classification

The International Classification of Headache Disorders (ICHD-II) was applied in the interview. The diagnoses were later reclassified according to ICHD IIIβ [16]. Those with secondary chronic headache exclusively due to medication overuse were excluded.

Chronic headache was defined as headache ≥15 days/months for at least 3 months or ≥180 days/year. Chronic post-traumatic headache (CPTH) included head and whiplash traumas. Cervicogenic headache (CEH) was classified according to the criteria of the Cervicogenic Headache International Study Group, requiring at least three criteria to be fulfilled, not including blockade of the neck due to the non-interventional nature of our study (Table 1) [17]. Headache attributed to chronic rhinosinusitis (HACRS) was defined according to the criteria established by the American Academy of Otolaryngology – Head and Neck Surgery (Table 2) adding that the symptoms had persisted for 12 weeks or more [18].

**Table 1:** Definition of cervicogenic headache [17]

Major criteria	I. Symptoms and signs of neck involvement Ia. Precipitation of head pain, similar to the usually occurring one: Ia1) by neck movement and/or sustained, awkward head positioning, and/or: Ia2) by external pressure over the upper cervical or occipital region on the symptomatic side. Ib. Restriction of the range of motion (ROM) in the neck. Ic. Ipsilateral neck, shoulder or arm pain of a rather vague, non-radicular nature, or – occasionally – arm pain of a radicular nature. II. Confirmatory evidence by diagnostic anaesthetic blockades. III. Unilaterality of the head pain, without sideshift.
Head pain characteristics	IV. Moderate-severe, non-throbbing pain, usually starting in the neck. Episodes of varying duration, or: fluctuating, continuous pain.
Other characteristics of some importance	V. Only marginal effect or lack of effect of indomethacin. Only marginal effect or lack of effect of ergotamine and sumatriptan. Female sex. Not infrequent occurrence of head or indirect neck trauma by history, usually of more than only medium severity.
Other features of lesser importance	VI. Various attack-related phenomena, only occasionally present, and/or moderately expressed when present: a) nausea, b) phono- and photophobia, c) dizziness, d) ipsilateral “blurred vision”, e) difficulties swallowing, f) ipsilateral oedema, mostly in the periocular area.

It is obligatory that one or more of the phenomena Ia–Ic are present

**Table 2:** Definition of rhinosinusitis by the American Academy of Otolaryngology Head and Neck Surgery [18]

*Major factors*	
Facial pain/pressure	
Nasal obstruction/blockage	
Nasal discharge/purulence/discolored postnasal drainage	
Hyposmia/anosmia	
Purulence in nasal cavity on examination	
Fever (acute rhinosinusitis)	
*Minor factors*	
Headache	
Fever (all nonacute)	
Halitosis	
Fatigue	
Dental pain	
Cough	
Ear pain/pressure/fullness	

Two major factors or one major and two minor factors are required for the diagnosis. Of note, facial pain requires another major factor associated with it for diagnosis, as facial pain plus two minor factors is not deemed sufficient for diagnoses of rhinosinusitis

#### Excessive daytime sleepiness

The ESS questionnaire describes eight daily situations in which the respondents estimate their likelihood of dozing off on a 0─3 scale, i.e. 0 = no chance, 1 = slight chance, 2 = moderate chance, 3 = high chance [14]. The ESS total scores (0─24) were dichotomized into scores ≤10 and >10; the latter is considered to be clinically significant EDS [19].

#### Statistical analysis

For descriptive data, proportions, means and SDs or 95% confidence intervals (CI) are given. We estimated binomial confidence intervals for proportions. Groups were compared using the *t*-test (continuous data) or the *χ* ^2^ test (categorical data).

Logistic regression models were used to evaluate presence of EDS as the dependent variable in secondary chronic headaches. Because of the low prevalence of EDS in the sample, we restricted the number of independent variables in multivariable analysis to three. All logistic regression models were estimated using penalized likelihood to reduce small-sample bias in maximum likelihood estimation [20, 21]. We conducted (1) bivariate analysis with type of chronic headache, i.e. CPTH/CER or HACRS, medication overuse (yes or no), and a propensity score (the propensity for having HACRS compared to CPTH/CER in a multivariable logistic regression model with age, sex, headache frequency, and concomitant migraine as independent variables), and (2) multivariable analysis forcing all three independent variables into the model. The results are presented with odds ratios (ORs) with 95% CIs.

Significance levels were set at *p* < 0.05, using two-sided test. All statistical analyses were performed using Stata version 14.2 (StataCorp, College Station, TX).

#### Ethical issues

The Regional Committee for Medical Research Ethics and the Norwegian Social Science Data Services approved the study. All participants gave informed consent.

## Results

In total 93 of the 113 eligible participants (82%) completed the ESS. Respondents and non-respondents to the ESS did not differ in age, gender composition or the distribution of headache diagnoses (data not shown).

A total of 36 people had CPTH, 19 people had CEH and 40 people had HACRS. Co-occurrence of CPTH and CEH was found in 7 people, while one person had co-occurrence of CPTH and HACRS. Six persons had other secondary chronic headaches, i.e. 3 post-craniotomy, 1 diving related, 1 pregnancy-related, and 1 post-meningitis.

Seven of those with CPTH, seven of those with CEH and ten of those with HACRS reported EDS, respectively. Only one of those with other secondary chronic headache reported EDS.

The people with CPTH and CEH were descriptive similar (gender, co-occurrence of migraine, medication overuse, ESS or EDS) and were merged for the purpose of statistical analyses. We included people with CPTH/CER and HACRS in the main analyses and excluded those seven persons with other secondary chronic headaches due to the low numbers and consequent statistical limitations.

The respondents with CPTH/CER had a higher proportion of subjects with headache frequency above the 75th percentile (≥90 headache days the past 3 months) and more severe disability than those with HACRS (Table 3).

**Table 3:** Descriptive statistics for respondents with chronic posttraumatic headache/cervicogenic headache vs. headache attributed to chronic rhinosinusitis. Number (%) unless stated otherwise

	Posttraumatic/cervicogenic headache (*N = 47*)	Rhinosinusitis headache (*N = 39*)	*p-value*
Age, mean (SD)	38.9 (4.2)	38.9 (3.8)	0.97
Gender			0.09
Female	34 (72)	34 (87)	
Male	13 (28)	5 (13)	
Education, highest attained			0.37
< 11 years	9 (19)	6 (15)	
11–15 years	27 (57)	25 (64)	
> 15 years	11 (24)	8 (21)	
Body mass index (kg/m^2^), mean (SD)	27.6 (5.3)^a^	25.5 (4.5)^b^	0.08
Daily smoker			0.61
No	16 (36)	16 (41)	
Yes	29 (64)	23 (59)	
Concomitant migraine			0.23
No	29 (62)	19 (49)	
Yes	18 (38)	20 (51)	
Medication-overuse			0.65
No	24 (51)	18 (46)	
Yes	23 (49)	21 (54)	
Number of headache days past 3 months			0.001
< 90 (Q1-Q3)	24 (53)	32 (89)	
≥ 90 (Q4)	21 (47)	4 (11)	
MIDAS score (grade)			0.038
0–20 (Little/no to moderate disability)	9 (22)	15 (45)	
> 20 (Severe disability)	31 (78)	18 (55)	
Epworth sleepiness scale score, mean (SD)	6.8 (4.5)	7.3 (4.2)	0.60
Excessive daytime sleepiness (ESS score > 10)			0.63
No	37 (79)	29 (74)	
Yes	10 (21)	10 (26)	

^a^ *n* = 36, ^b^ *n* = 35

The overall prevalence of EDS was 23% (95% CI 16─33) among those with CPTH, CER or HACRS; 22% (95% CI 14─33) among women and 28% (95% CI 13─51) among men (Table 4). The prevalence of EDS in CPTH/CER without versus with co-occurrence of migraine was 24% (95% CI 12─42) and 17% (95% CI 6─39), respectively (*p* = 0.54). The prevalence of EDS in HACRs without versus with co-occurring migraine was 16% (95% CI 6─38) and 35% (95% CI 18─57), respectively (*p* = 0.17).

**Table 4:** Prevalence (%) of excessive daytime sleepiness (ESS >10) in people with secondary chronic headache

	Posttraumatic/ cervicogenic headache		Rhinosinusitis headache		Total	
	N	Prevalence (95% CI)	N	Prevalence (95% CI)	N	Prevalence (95% CI)
Men	13	30.8 (12.7 to 57.6)	5	20.0 (3.6 to 62.4)	18	27.8 (12.5 to 50.9)
Women	34	17.6 (8.3 to 33.5)	34	26.5 (14.6 to 43.1)	68	22.1 (13.8 to 33.3)
All	47	21.3 (12.0 to 34.9)	39	25.6 (14.6 to 41.1)	86	23.3 (15.6 to 33.2)

Headache diagnosis, medication overuse or the composite propensity score (age, gender, headache frequency and co-occurring migraine) was not associated with EDS in bivariate or multivariable analysis (Table 5).

**Table 5:** Odds for having EDS, defined as ESS > 10. Penalized maximum likelihood logistic regression

		Bivariate (*n* = 81–86)				Multivariable (*n* = 81)		
Covariate	*n*	Odds ratio	95% CI	*P*	*n*	Odds ratio	95% CI	*P*
Headache type								
Posttraumatic/cervicogenic^a^	47	1			45	1		
Rhinosinusitis	39	1.27	(0.48 to 3.39)	0.63	36	1.71	(0.55 to 5.35)	0.35
Medication-overuse								
No^a^	42	1			41	1		
Yes	44	0.44	(0.16 to 1.21)	0.11	40	0.44	(0.16 to 1.25)	0.12
Propensity score (age, sex, headache frequency, migraine)	81	0.77	(0.07 to 8.33)	0.83	81	0.35	(0.02 to 5.53)	0.45

^a^Reference category

Applying the *χ* ^2^-test in non-adjusted analyses, no significant differences (data not shown) were found in those with and without EDS depending on socio-demographics, body mass index, smoking, alcohol, caffeine, other sleep disorders, anxiety/depression, comorbidity of other disorders or medication use for other conditions.

## Discussion

In this large population-based study almost one out of four subjects with secondary chronic headache reported EDS. The main finding was that the prevalence of EDS did not differ between those with CPTH/CEH and HACRS. Furthermore, medication-overuse, or the composite propensity score (age, gender, headache frequency and co-occurring migraine) was not associated with EDS in this population.

No previous study has investigated EDS for secondary chronic headache in the general population. The prevalence of EDS in men and women with secondary chronic headache corresponds to that for people with primary chronic headache in the general population (20.6% among women, 22.5% among men) and is comparable to data reported from the Norwegian general population (16.1% among women, 20.1% among men) [11, 22]. Here, we did not include a directly comparable headache-free control group and caution is therefore warranted in this comparison. Also, due to the limited sample size in the present study, the risk of type 2 errors must be considered.

Little is known about the precise relationship between headache and sleep problems, when these occur concurrently. An association between EDS and different pain conditions has been reported [2, 3]. Pain may disturb sleep and give rise to EDS, but sleep loss and EDS may also contribute to pain. Some of these secondary headaches are poorly understood, thus, further research is warranted [23]. Studies suggest that CEH can be explained by local factors in the neck with dysfunction of the neck muscles and mechanical cervical spine pathology leading to limited cervical movements and projection of the pain [24]. Headaches attributed to head trauma and whiplash trauma have instead been suggested to represent an interplay between the physical injury, neuroinflammation, psychological disturbances and emotional stress of the accident [25, 26]. Finally, longstanding oedema of the nasal mucosa and rhinosinal inflammation result in chronic rhinosinusitis which may give chronic headache [12]. The present study reported that CPTH/CEH and HACRS had similar prevalence of EDS despite these different headache forms probably being caused by different pathophysiological mechanisms. Therefore, it may be the complex burden of pain, more than the specific condition that is associated with EDS. Furthermore, the prevalence was comparable to that of two other different headache entities; chronic migraine and chronic tension-type headache [11].

The population-based sample in the present study was large, and the high response rate should ensure that the sample was representative of the general population. Even though the sample size of secondary chronic headache may seem small, this is the largest sample of subjects with secondary chronic headache recruited from the general population.

The diagnostic criteria of CEH and HACRS have been discussed for many years [12, 27]. When the study was conducted the more vague ICHD-II criteria for CEH were in use and HACRS was not recognized as a cause of chronic headache. Thus, to improve the diagnostic accuracy we used supplementary definitions. All patients diagnosed with CEH or HACRS in the present study fulfil the new ICHD-IIIβ criteria for these chronic headaches.

Face-to-face interviews by headache experts, as in the present study, provide more valid headache diagnoses than questionnaire-based studies [28]. The ESS is a widely used, validated questionnaire for evaluating subjective daytime sleepiness, and the score is associated with clinically important outcomes, such as cognitive impairment, cardiovascular mortality, and injuries [29, 30].

The 30–44 years age range in our study was chosen in order to ascertain a general population sample without much co-morbidity of non-headache disorders. Because EDS varies with age, our findings may not be generalizable to younger or older populations. The study lacked data on sleep quality and sleep duration that may be associated with sleepiness in headache [5, 6, 8, 9].

The overall sample size limited the number of variables that could be analyzed as potential confounders, and this also lead us to dichotomize many variables for use in the analyses. Also, due to the small number in the present study, the risk of type 2 errors must be considered. Finally, the cross-sectional design in the present study does not permit any conclusions about causality.

In conclusion, there was no difference in the prevalence of EDS between subgroups of different secondary chronic headache diagnoses.
